# Retrospective Observational Study to Assess Safety and Tolerability of Nebulized Colistin for the Treatment of Patients With Pneumonia in Real-World Settings in Respiratory ICU

**DOI:** 10.7759/cureus.54652

**Published:** 2024-02-21

**Authors:** Deepak Talwar, Deepak Prajapat, Surbhi Talwar, Dhruv Talwar

**Affiliations:** 1 Pulmonary, Sleep, and Critical Care Medicine, Metro Centre for Respiratory Diseases, Noida, IND; 2 Pulmonary and Critical Care Medicine, Metro Centre for Respiratory Diseases, Noida, IND; 3 Nephrology, University Hospitals Coventry and Warwickshire (UHCW), Coventry, GBR; 4 Medicine, Jawaharlal Nehru Medical College, Datta Meghe Institute of Medical Sciences (Deemed to be University), Wardha, IND

**Keywords:** respiratory tract infection, polymyxin, nebulizer, pneumonia, critical care

## Abstract

Introduction: Colistin is used to treat hospital-acquired pneumonia and ventilator-associated pneumonia. However, direct drug deposition at the site of infection may improve its efficacy and reduce systemic exposure. The aim of this study was to assess the safety and tolerability of nebulized colistin among Indian patients diagnosed with pneumonia caused by multidrug-resistant gram-negative bacilli in real-world settings.

Methodology: We retrospectively reviewed the medical records of patients treated with nebulized colistin for pneumonia. We assessed the adverse events and relevant abnormal laboratory findings of nebulized colistin therapy.

Results: All enrolled patients (N=30, males: 22, females: 8; average age: 71.06 years) were treated for 13.36 days. Almost 80% of patients had a history of shortness of breath, which was a major symptom when they were admitted to the hospital. The patients were administered nebulized colistin for an average of six days (8 hours per day). The most common dosing schedule was 1 million international units (MIU)/8 hours. No serious adverse event was observed, and only one patient died while on the treatment but the death was not related to colistin treatment. The average sequential organ failure assessment score for all patients was 6.5.

Conclusion: Our study demonstrated the efficient clinical utility and well-tolerated safety profile of nebulized colistin in the treatment of patients with pneumonia. Neurotoxicity and nephrotoxicity were not reported. Since a significant percentage of patients were with chronic respiratory diseases, our study further indicates the safety and effectiveness of nebulized colistin in chronic obstructive pulmonary disease (COPD) patients too.

## Introduction

Pneumonia is a severe form of acute lower respiratory infection caused by a broad spectrum of pathogens. Its mortality was estimated to be 1.575 million globally and 0.371 million in India [1]. It was also the leading cause of mortality among children and an estimated 808,694 deaths in children (<5 years old) with pneumonia were reported in 2017, accounting for 15% of all deaths in children who are <5 years old [2].

Pneumonia can be community-acquired (outside the hospital) or infection-acquired during a hospital stay. Hospital-acquired pneumonia (HAP) and ventilator-associated pneumonia (VAP) caused by multidrug-resistant (MDR) gram-negative bacteria such as *Pseudomonas aeruginosa*, *Acinetobacter baumannii*, *Klebsiella pneumoniae,* or methicillin-resistant *Staphylococcus aureus* (MRSA) strains have high rates of recurrence and develop resistance to antibiotics [3,4]. Limited therapeutic options and a lack of novel therapeutics in the pipeline forced clinicians to reconsider the applications of colistin (a polymyxin antibiotic) which was discovered >50 years ago as a salvage therapy against these microbes [4-7].

Intravenous (IV) administration of colistin to treat pneumonia, caused by MDR bacteria, is challenging due to its poor alveolar penetration [8,9]. Further, IV administration in patients with pneumonia was often associated with nephrotoxicity and neurotoxicity [10,11]. Nebulization of antibiotics provides direct drug deposition to the site of infection while curtailing systemic exposure [12]. This increases antimicrobial efficacy and minimizes drug resistance and colistin-induced nephrotoxicity and neurotoxicity [3,11]. However, the underlying lung diseases may affect the safety and efficacy of the nebulized drug therapy [13].

Most real-world data available report the use of nebulized colistin in treating patients with cystic fibrosis (CF) [14,15]. However, at present, very limited evidence is available on the safety of nebulized colistin for the treatment of MDR nosocomial pneumonia patients in Indian routine clinical care settings. Our pilot study aimed to assess the safety and tolerability of nebulized colistin in Indian patients diagnosed with pneumonia caused by gram-negative bacteria in real-world settings. The secondary objective was to evaluate the utilization pattern and clinical outcomes of nebulized colistin in patients with pneumonia. This real-world data will provide valuable insight to make informed treatment decisions.

## Materials and methods

Study design

This post-marketing, observational, non-interventional pilot study retrospectively reviewed the medical records of patients with pneumonia at Metro Hospitals, Noida, New Delhi. Patients aged ≥18 years, diagnosed with HAP or VAP caused by MDR gram-negative bacteria (i.e., *Acinetobacter baumannii*, *Pseudomonas aeruginosa*, etc.) and who were treated with nebulized colistin therapy for at least ≥2 days were included in this study. The dosage was given at the physician’s discretion. The patients treated with nebulized colistin for <2 days, those with a history of asthma/COPD, and those who were on continuous renal replacement therapy at baseline were excluded.

The study was conducted in accordance with the Declaration of Helsinki. The study was approved by an institutional ethics committee.

Study variables

Patient data from medical records such as demographics, clinical characteristics, medical history, microbiological testing, comorbid conditions, colistin dosing and duration, type of nebulizer, concomitant therapy, clinical outcomes, safety and tolerability, clinical outcomes post-treatment or discharge were collected using a predesigned case report form (CRF). This study was descriptive in nature; thus, no control or comparator groups were included. 

Data reported by the physician from the CRF were transferred to a single Excel-based patient database. All data were de-identified and anonymized. Incomplete data were excluded from analysis following discussion with the investigator.

Outcome assessments

Primary outcomes measured in this study included adverse events and relevant abnormal laboratory findings (serious/non-serious, expected/unexpected, and related/non-related), percentage of patients with adverse events, treatment discontinuation rates (early discontinuation or discontinuation due to adverse events), and adverse events of special interest (bronchospasm, nephrotoxicity, or neurotoxicity). Nephrotoxicity was defined using the RIFLE (Risk, Injury, Failure, Loss of kidney function and End-stage kidney disease) classification. Exploratory outcomes measured included nebulized colistin utilization pattern - dosing, duration, nebulizer type, concomitant treatment, etc.

Statistical analysis

Descriptive analyses were conducted with STATA version 15.1 (College Station, TX: StataCorp LLC). Numerical variables were described using the number of observations (n), mean and standard deviation, minimum, maximum, and median. Missing data were excluded on a case-by-case basis and were not imputed.

## Results

Of all 30 patients enrolled in this study, 22 (73%) were males and 8 (27%) were females. The average age was 71.06 years and most patients were 65-80 years old. Patients were treated for four to 34 days and most were treated for 9 to 15 days (average treatment days: 13.36) (Table 1).

**Table 1:** Baseline characteristics N=number of patients.

Study Population	N (%)
Total	30
Male	22 (73%)
Age (mean)	71.06 years
Treatment days (mean)	13.36 days
Reason for hospitalization	
Respiratory failure	13 (43%)
COPD	13 (43%)
Sepsis	8 (26%)
Hypertension	9 (30%)
Shortness of breath	8 (26%)
Diabetes	5 (16%)
Need for ventilator support	7 (23%)

Around 80% (24/30) of patients reported a history of shortness of breath, and difficulty in breathing was the major symptom at the time of hospitalization. Other common symptoms included COPD/asthma (11/30), coughing (20/30), wheezing (12/30), drowsiness, swelling in limbs, hypertension, high fever, and sore throat (Table 2).

**Table 2:** Symptoms of patients *Other symptoms include wheezing (12/30), sputum (9/30), drowsiness, swelling in limbs, hypertension, high fever, and sore throat. N=number of patients.

Symptoms	N (%)
History of COPD/asthma	11 (36%)
Coughing	20 (66%)
Shortness of breath	24 (80%)
Difficulty breathing	24 (80%)
Other symptoms*	20 (66%)

Major reasons behind hospitalization included respiratory failure (13/30), COPD (13/30), sepsis (10/30), hypertension (9/30), shortness of breath (8/30), diabetes (5/30), and bronchiectasis (5/30). Overall, 23% of patients needed ventilator support, of which 85% were provided with non-invasive mechanical ventilators and 15% were given invasive mechanical ventilation (Table 1).

Respiratory rates of patients ranged between 16 and 41 breaths per minute (bpm) with an average rate of 23 bpm, and 63% of patients had respiratory rates above 20 bpm. The systolic blood pressure of patients ranged between 90 and 189 mm Hg (average: 124 mm Hg). The diastolic blood pressure of patients ranged between 50 and 100 mm Hg (average: 76 mm Hg). Only 33% of patients who were admitted had a prior medical history.

Nebulized colistin treatment

Patients were administered nebulized colistin for an average of six days (8 hours per day). It was prescribed for a maximum of 12 days and a minimum of two days. The most common dosing schedule was 1 MIU/8 hours but a few patients were also given 1 MIU/12 hours (n=3) (Table 2).

Most patients were prescribed some other inhaler such as Duolin - ipratropium (20 mcg) + levosalbutamol (50 mcg), Budamate - budesonide (200 mcg) + formoterol (6 mcg), Nebzmart - budesonide 0.5 mg/2ml, Flohale - fluticasone propionate, and Formonide - budesonide (200 mcg) + formoterol (6 mcg) before being prescribed colistin.

Adverse events

Among all patients who were administered colistin treatment, no serious adverse event was observed, and only one patient died while on the treatment but the death was not due to colistin treatment. Moreover, no patient experienced bronchospasm during the study. Neurotoxicity and nephrotoxicity were not reported for any of the included patients.

Acute Physiology and Chronic Health Evaluation (APACHE)-III score

The APACHE-III score was available only for three patients. APACHE score was 4 in two patients and it was 19 in one patient on day 2.

Sequential organ failure assessment (SOFA)

The SOFA score was available only for four patients (Patient 001, Patient 006, Patient 012, and Patient 014). The average SOFA score among all these patients was 6.5.

## Discussion

This retrospective pilot study demonstrated that nebulized colistin is safe and tolerable in patients with MDR gram-negative bacilli pneumonia. Neurotoxicity and nephrotoxicity were not observed in any of the enrolled patients. However, one patient died while on the treatment but the death was not due to colistin treatment. Our findings are comparable with previous studies that demonstrated the safety and tolerability of nebulized colistin therapy [3,11,16-19]. A previous study demonstrated that prophylaxis with inhaled colistin improved ICU survival in patients with VAP [20].

The rationale backing the use of nebulized colistin therapy is to maximize drug delivery to lung alveolar tissues and limit the potential for systemic side effects such as nephrotoxicity and neurotoxicity [10]. As nebulization of antibiotics targets airway and lung parenchyma, it results in higher drug deposition in the infected lung parenchyma with concentrations above the minimum inhibitory concentrations (MICs) for most gram-negative pathogens [21,22]. Furthermore, the absorption, efficacy, and antimicrobial efficacy of the nebulized antibiotic were demonstrated to be higher compared to its IV administration and are paired with low systemic absorption [8]. In a pharmacokinetic study of aerosolized colistin in patients with CF, the maximum observed serum colistin level was 0.178 mg/L, which was much lesser than the maximal level (3.6-13.2 mg/L) attained by the IV route, and it was undetectable until 24 hours post inhalation [14,23]. Though we did not measure plasma colistin levels, it is reasonable to assume that low plasma levels of colistin through nebulization led to lower systemic side effects in our patient’s cohort. Clinical practice guidelines by the Infectious Diseases Society of America (IDSA) and the American Thoracic Society recommended using inhaled colistin in addition to the IV route for treating pneumonia caused by MDR gram-negative pathogens [24].

Though several studies evaluated the efficacy and safety of nebulized colistin in patients with MDR gram-negative bacilli pneumonia across the world, uncertainty around the risk of nephrotoxicity and neurotoxicity associated with colistin limits the generalizability of the results of these studies to Indian patients. This study confirmed the safety and clinical utility of nebulized colistin in Indian patients with no cases of nephrotoxicity and/or neurotoxicity.

Another important finding of our study was that the majority of patients had underlying COPD. Treatment with nebulized colistin in patients with COPD has been demonstrated to reduce the number and duration of hospitalizations due to COPD exacerbation. Although RICU settings limited the clinical background of patients in our study with the majority having COPD, the results indicate excellent tolerability and safety in patients with underlying lung diseases [25]. As a retrospective study, the results of this study need to be confirmed by a randomized clinical trial with a large patient population.

## Conclusions

Nebulized antibiotic therapy offers high concentrations of antibiotics directly to the site of lung infection and is associated with low systemic exposure. Nebulized colistin was shown to be an effective therapeutic treatment for pneumonia patients in this trial. Additionally, the trial showed acceptable safety, with no reports of neurotoxicity or nephrotoxicity for any of the pneumonia patients who were part of the study. Nonetheless, additional research on nebulized colistin may offer a more optimistic picture of nebulized antibiotics in the future.
